# Understanding of Medical Students' Information Needs in Emergency Cases: The Implications for Emergency Management in Teaching Hospitals of Iran

**Published:** 2011-01-01

**Authors:** M Kahouei, R Eskrootchi, F Ebadi Fard Azar

**Affiliations:** 1 Department of Health Information Technology,Para-Medical and Nursing School, Semnan Universityof Medical Sciences, Semnan, Iran; 2 Department of Librarian, Management and Medical Informatics School, Tehran, Iran; 3 Department of Health, Iran University of Medical Sciences, Tehran, Iran

**Keywords:** Medical, Students, Information, Needs, Emergency

Dear Editor,

The practice of emergency medicine is the ability to perform indicated clinical procedures in a skillful and safe manner.[1][2] Due to multiple factors, including the unpredictable nature of emergency medicine, clinical experience may be quite variable. So clinical experience in the emergency department (ED) is the foundation of emergency medicine.[3][4][5][6] In teaching hospitals, clinical encounter is an increasing significant component of medical students' curriculum [7][8] and one of the clinical experiences of medical students is emergency medicine.[9][10] Thus, all students who are graduated from medical schools should be capable of handling emergency situations.[11][12] While the clinical encounter is understood to be a significant educational experience, little is known about the information of medical students in response to clinical problems, specially in emergency cases. To date, many studies address physicians' information needs but few authors report studies describing medical students' information needs in the ED. The present study aims at analyzing the information needs of medical students in emergency departments which differs from most of the past studies by focusing on the information needs of students, rather than those of clinicians. This study seeks to provide a better understanding of students' information needs and analyzes the content of questions of medical students in response to a clinical encounter that can impact on the clinical education quality, the recognition of students' educational needs, and the educational improvement in emergency and better management of patients by medical students that decreases patient's stay and promotes patient's satisfaction.

The information needs of medical students in the emergency departments are evaluated at teaching hospitals in Iran University of Medical Sciences. A questionnaire was used. During observations, questions were asked by the medical students. In the questionnaire, following the review of the literature, a survey instrument was developed including 17 questions primarily related to the medical students' demographics data, and rate of information needs. Two sets of categories were developed to classify: 1) The content of the information needs recorded as questions and 2) the information need rate. The categorization of students' questions was based on the content of each question; such as diagnosis, treatment and organization. The categorization of information need rate was based on little, moderate and high.

Seventy medical students participated in the study while 45 (64%) were male. 350 questions were noted during our observations. 147 (42%) questions pertained to diagnosing the patient's problem. Organizational questions such as hospital policies and procedures made up a less portion of the questions asked by the students. The analysis revealed a statistical significant relationship (r-0.576, p<0.01) among the students' diagnostic and therapeutic questions. The students reported that the majority of their information needs were laboratories (n=59, 84.3%) and radiography results (n=52, 74.3%) and the least of them were medicolegal coordination (n=7, 10%). The Chi-square analysis revealed a statistical significant relationship among need rate to radiography, laboratory and drug treatment results (p<0.01).

Like those of experienced clinicians, students' information needs most often pertained to diagnosing a problem or choosing a treatment. Cogdill and Moore examined the first-year medical students' information needs in response to a clinical scenario. They found that the majority of students questions asked were related to diagnosis and treatment.[7] The observation showed students' questions were often asked because of a breakdown in the information flows. Although some questions were asked to elicit opinions or confirm order, many questions were asked because the students did not receive the needed information to make or implement a decision. Only 24% (n=84) of the questions noted during observations related to hospital policies, procedures, coordination and management issues. In emergency cases the answer to the organizational questions enabled the students to function more effectively and keep the unit running smoothly.[13] The low occurrence of organizational questions in the students indicates a few students had understood the importance of the interrelationship between clinical and organizational aspects of work in clinical units. This study indicated that 42% questions pertained to diagnosis thus students must have high need to these results because they help to the students to work out the diagnosis. The results revealed a significant correlation among need rate to radiography, laboratory and drug treatment results. In other words, those who had higher need to laboratory and radiography results also had higher need to drug treatment results. The opposite was true for those who had low need to laboratory and radiography results. This study showed that medicolegal issues were not important for the majority medical students, as only 10% (n=7) of students reported that they had high need to this information, since medicolegal affairs are subsequences that some of the patients who are referred to the ED, thus it is important that medical students increasingly consider to them.

Educational programs aiming to promote the medical students' awareness about organizational information and medicolegal issues must be provided in medical schools.
